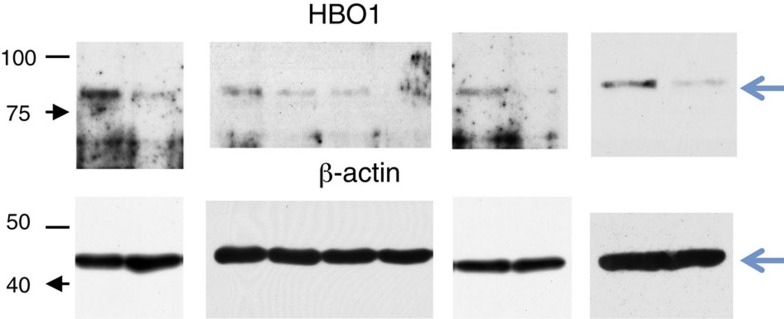# Author Correction: Phosphorylated HBO1 at UV irradiated sites is essential for nucleotide excision repair

**DOI:** 10.1038/ncomms16214

**Published:** 2018-04-20

**Authors:** Hiroyuki Niida, Ryoichi Matsunuma, Ryo Horiguchi, Chiharu Uchida, Yuka Nakazawa, Akira Motegi, Koji Nishimoto, Satoshi Sakai, Tatsuya Ohhata, Kyoko Kitagawa, Shinichi Moriwaki, Hideo Nishitani, Ayako Ui, Tomoo Ogi, Masatoshi Kitagawa

Nature Communications 8: Article number: 16102; DOI: 10.1038/ncomms16102 (2017); Published online 07 18 2017; Updated 04 20 2018

The original version of this Article contained an error in Fig. 1b, in which the cells were incorrectly labelled as ‘HeLa’, rather than the correct ‘HEK293’

Also, the following was omitted from the end of the legend of Fig. 1b: ‘Scanned images were subjected to linear adjustments of light–dark and contrast by Microsoft Power Point.’

These have been corrected in both the PDF and HTML versions of the Article.

The original version of the Supplementary Information associated with this Article contained errors in Supplementary Figs 3d, 5c, 7 and 9.

In Supplementary Fig. 3d, the shHBO1 image labelled ‘600’ inadvertently repeated those labelled ‘450’.

In Supplementary Fig. 5c, the images labelled ‘98BR’, ‘3KA’ and ‘7HM’ were previously incorrectly showing 7HM, NMKW and 48BR cell lines, respectively. The correct version of Supplementary Fig. 5c appears below as Fig. 1:

which replaces the previous incorrect version, which appears below as Fig. 2:

In Supplementary Fig. 7, the image displayed the results of one experiment instead of three independent experiments as indicated in the legend. The correct version of Supplementary Fig. 7 appears below as Fig. 3:

which replaces the previous incorrect version, which appears below as Fig. 4:

In Supplementary Fig. 9, the uncropped images for Fig. 1b below the labels ‘pS50/53-’ and ‘pS50/53 UV’ were swapped, as were those below the labels ‘HBO1 –’ and ‘HBO1 UV’. Also, the blue arrow indicating the HBO1 band was missing from the image labelled ‘HBO1 –’.

In Supplementary Fig. 9, in the uncropped images for Fig. 2b, the bottom-left image labelled ‘HBO1’ previously incorrectly presented with an unrelated uncropped blot and the bottom-right image for beta actin incorrectly presented from a different experiment. The correct version of the uncropped images for Fig. 2b given in Supplementary Fig. 9 appears below as Fig. 5:

which replaces the previous incorrect version, which appears below as Fig. 6:

In Supplementary Fig. 9, in the uncropped images for Supplementary Fig. 5c, the third image from the left under the label ‘HBO1’ was previously incorrectly showing 48BR cell lines, rather than the intended 7HM cell lines. The correct version of the uncropped images for Supplementary Fig. 5c given in Supplementary Fig. 9 appears below as Fig. 7:

which replaces the previous incorrect version, which appears below as Fig. 8:

The HTML has been updated to include a corrected version of the Supplementary Information.

## Figures and Tables

**Figure 1:**
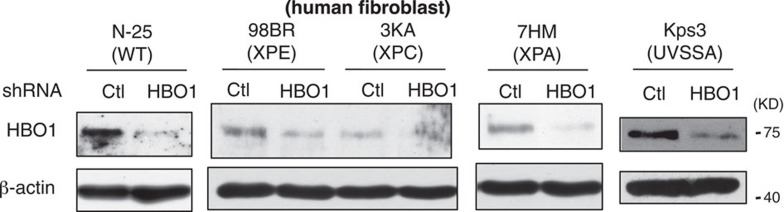


**Figure 2:**
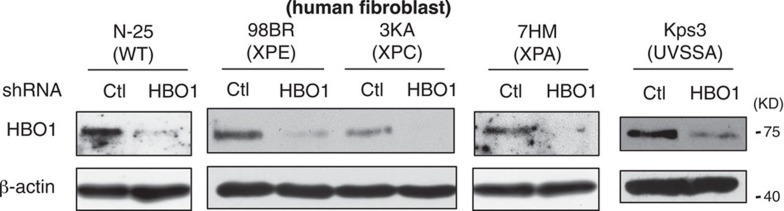


**Figure 3:**
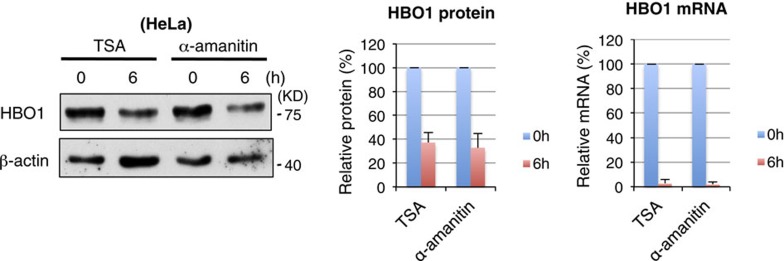


**Figure 4:**
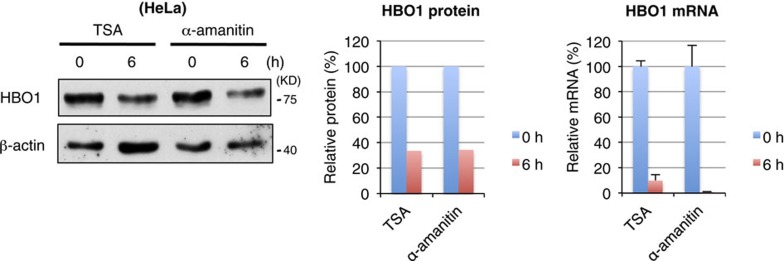


**Figure 5:**
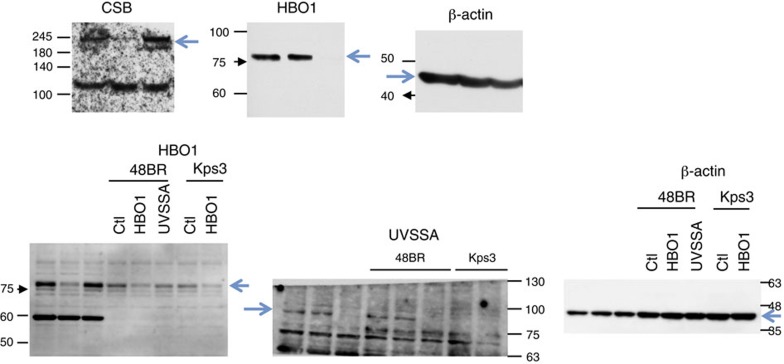


**Figure 6:**
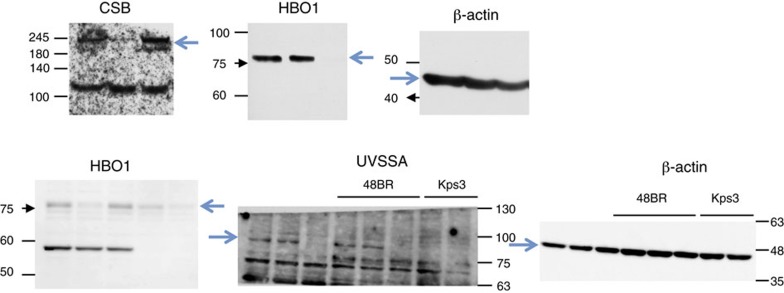


**Figure 7:**
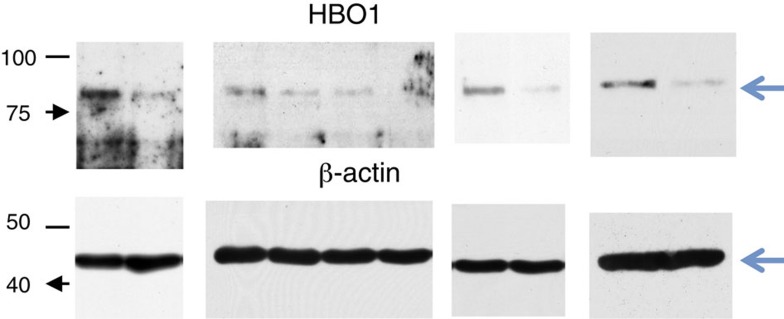


**Figure 8:**