# Supplementary material for: Phosphorylated HBO1 at UV irradiated sites is essential for nucleotide excision repair
Source: Nat Commun. 2017 Jul 18;8:16102. doi: 10.1038/ncomms16102 (PMC5520108; doi:10.1038/ncomms16102)
Supplement: Supplementary Information [file ncomms16102-s1.pdf]

Supplementary Figure 1

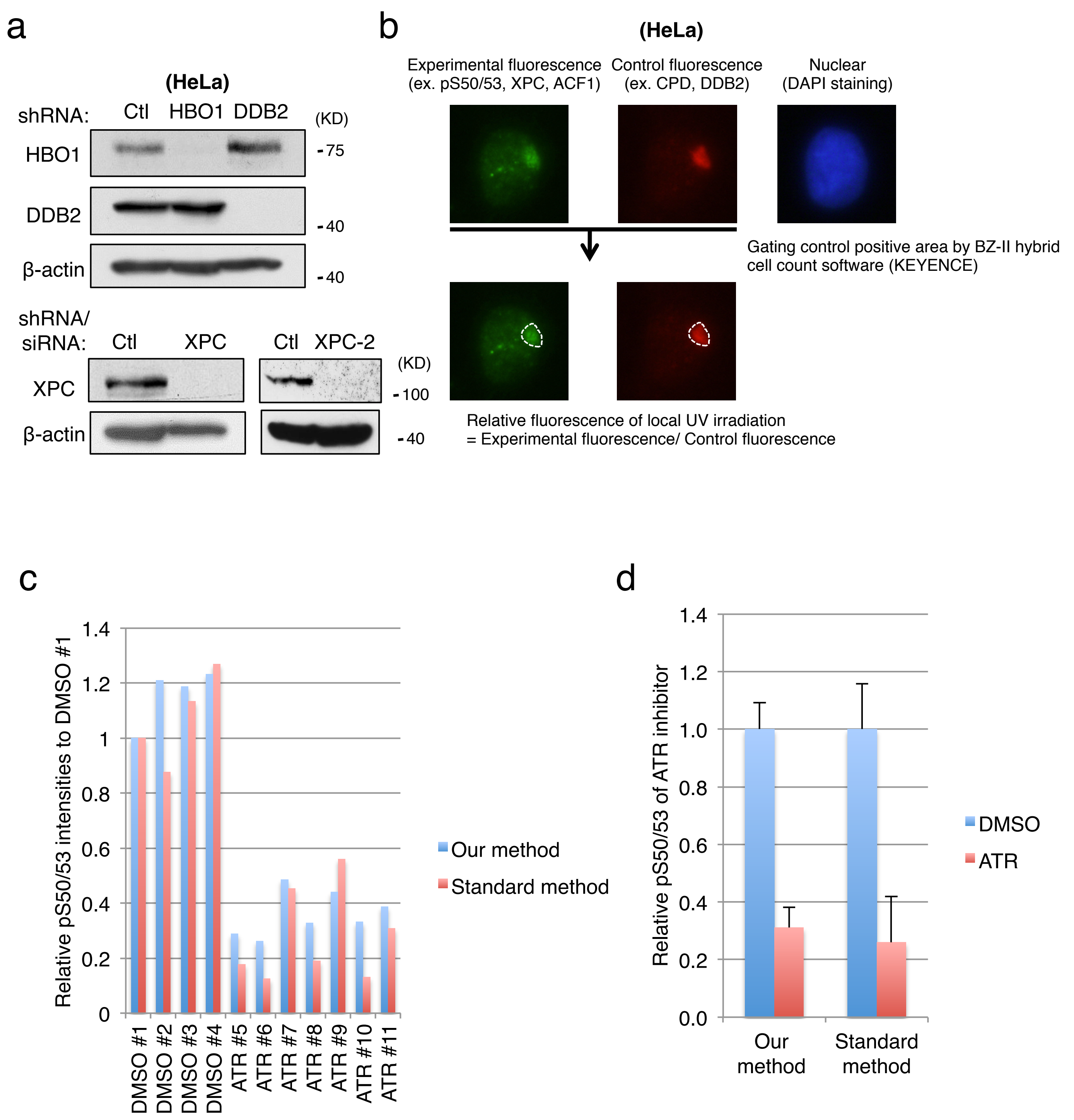

e

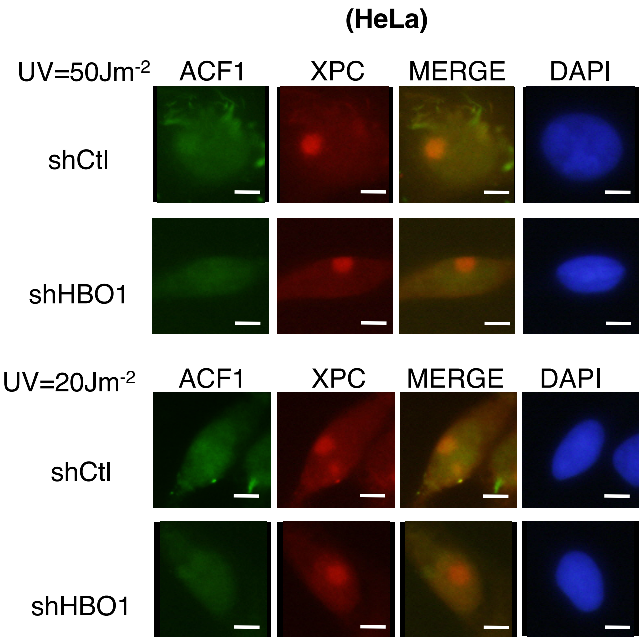

f

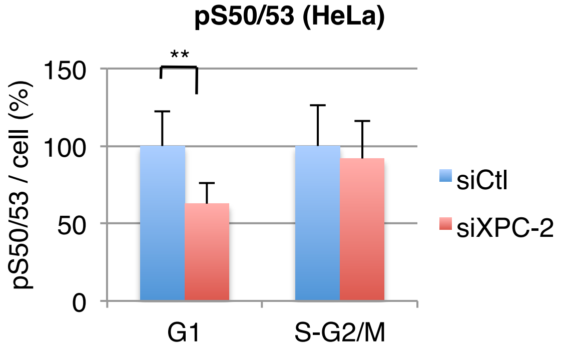

Supplementary Fig. 1 HBO1, DDB2 and XPC depleted HeLa cells.
(a) ShHBO1 and shDDB2 stably expressing HeLa cells were established as described previously[15]. To establish stably expressing shXPC HeLa cells, HeLa cells were transfected with XPC shRNA plasmid. At 48 h after transfection, cells were selected with 10 μM puromycin for 3 days and then cultured in normal medium for 7 days. At least 24 colonies were isolated, and XPC expression was determined by western blotting. SiXPC-2 transfected shCtl cells were used for experiments after 48 h transfection. (b) Definition of relative fluorescence at local UV irradiated sites. To calculate the relative fluorescence at local irradiated sites, the control fluorescence (CPD, DDB2) positive area was gated by BZ-II hybrid cell count software (KEYENCE) and fluorescence was quantified. Then, the fluorescence of the factor in question (pS50/53, XPC, ACF1) at the same area was quantified. Relative fluorescence was calculated as the experimental fluorescence divided by the control fluorescence. (c, d) Comparison of relative pS50/53 fluorescence calculated by our method and standard method for individual cell (c) or combined cells (d). Relative pS50/53 intensity by our method was calculated as follows. Local UV irradiated area was identified by DDB2 and relative intensity of pS50/53 HBO1 at UV lesion sites was expressed as ratio of pS50/53 HBO1 to DDB2 fluorescence intensity at the damage site. Relative pS50/53 intensity by Standard method was calculated as follows. Local UV irradiated area was identified by DDB2 staining. The relative intensity of pS50/53 HBO1 at UV lesion sites was expressed as the ratio of fluorescence signal intensity at the damage site to the respective intensity in the remaining nuclear area. DMSO: control, ATR: ATR inhibitor treated. Both methods provided very similar results for single cells (c) and combined cells (d). (e) ACF1 and XPC accumulated at local-UV 50 and 20 Jm^{-2} irradiated sites of MeOH/Acetone fixed cells. ACF1 accumulated at DNA lesions in shCtl but not shHBO1 cells. ShCtl and shHBO1 cells were local irradiated with 50 and 20 Jm^{-2} UV and incubated for 30 min. Cells were co-immunostained with anti-ACF1 and anti-XPC antibodies. Bars, 5 μm. (f) Phosphorylation of HBO1 Ser50 and Ser53 in G1 phase is dependent on XPC. HeLa cells transfected siXPC-2 was used for this experiment. Cells were irradiated with 50 Jm^{-2} UV and incubated for 30 min. Cells were immunostained with anti-pS50/53 antibody. DNA was stained with DAPI. DNA contents and fluorescence of pS50/53 in each cell were quantified by the IN CELL Analyzer. Cells in G1 or S-G2/M phase were defined by DNA content. Average of pS50/53 intensity in G1 and S-G2/M phase of siXPC-2 cells was shown as % of siCtl cells. n=1400, error bars indicate means ± S.D. **P<0.01 (Student's t test).

Supplementary Figure 2

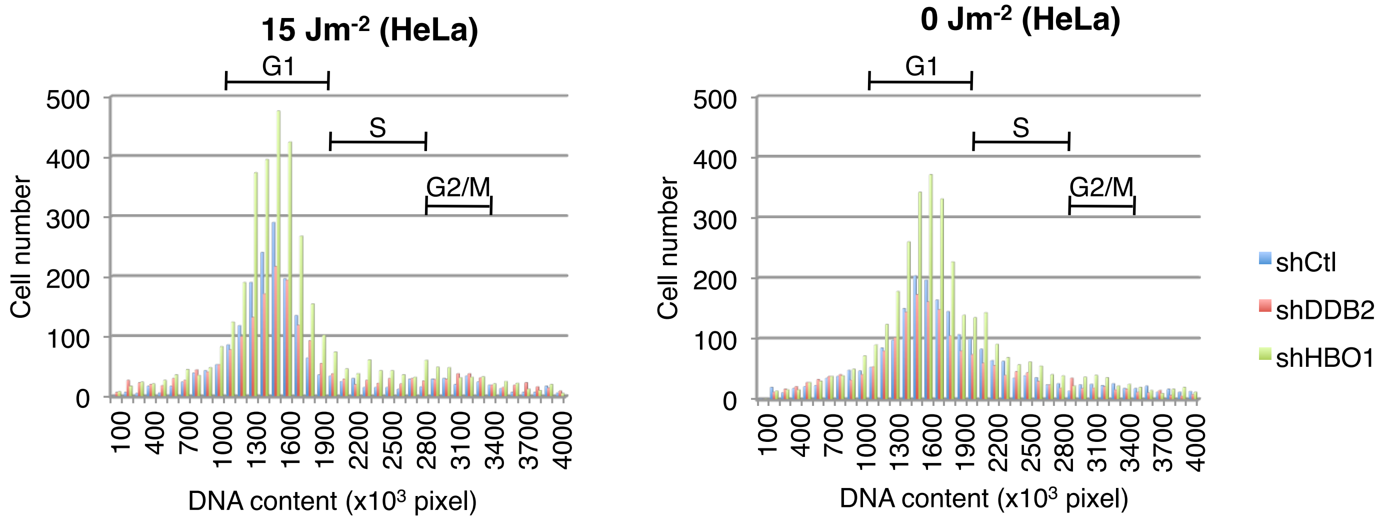

Supplementary Fig. 2 Cells prepared for UDS assay (related to Fig. 2).
Cells were treated with 50 ngml $^{-1}$nocodazole for 15 h and then washed three times with PBS to release cells from M phase. Cells were synchronized after 3 h by releasing from M phase and irradiated with mock (right) or 15 Jm^{-2} UV (left). After UV irradiation, cells were treated with 5-ethynyl-2'-deoxyuridine (EdU) for 3 h. After EdU incorporation, DNA contents of synchronized cells were measured by staining with Hoechst 33342 to gate G1 cells. UDS of each cell was measured by relative fluorescence of EdU in the gated cells.

Supplementary Figure 3

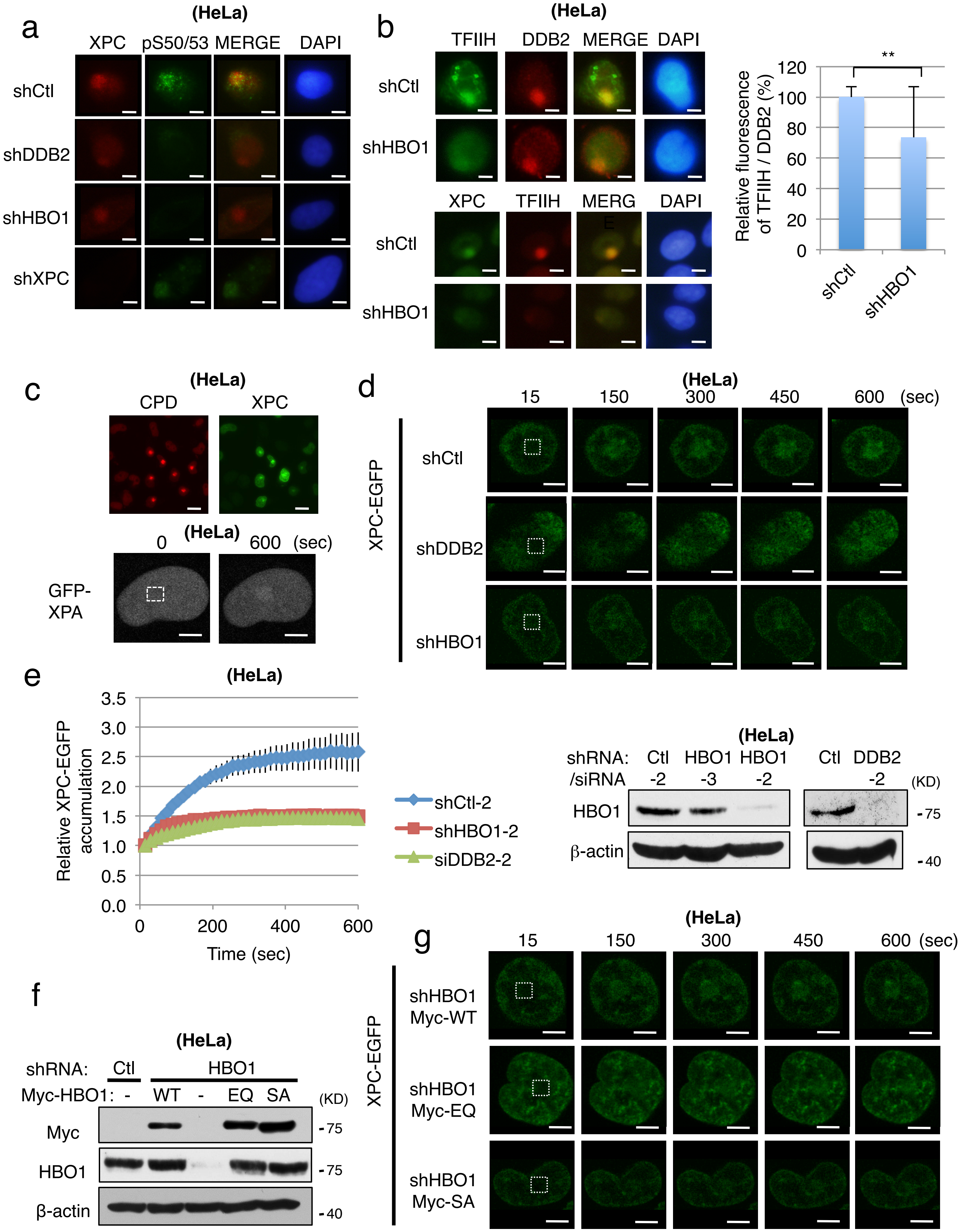

Supplementary Figure 3 continued

Supplementary Fig. 3 Accumulation of XPC-EGFP in 405-nm laser-irradiated areas
(a) Diminished pS50/53 signal in shDDB2 HeLa cells. ShCtl, shDDB2, shHBO1 and shXPC HeLa cells were local irradiated with 50 Jm^{-2} UV and cultured for 30 min. Cells were co-immunostained with anti-pS50/53 and anti-XPC antibodies. Bars, 5 μm. (b) Accumulation of TFIIH and DDB2 at local UV irradiated sites in shHBO1 HeLa cells. ShCtl and shHBO1 cells local irradiated with 50 Jm^{-2} UV were cultured for 30 min. Cells were co-immunostained with anti-TFIIH and anti-DDB2 or anti-XPC antibodies. Definition of relative fluorescence is shown in Supplementary Fig. 1b. The relative TFIIH/DDB2 intensity of shHBO1 cell is shown as % of shCtl cells. Bars, 5 μm. n=50 cells from three independent experiments; error bars indicate means ± S.D. ***P*<0.01 (Student's t test). (c) The 405-nm laser with Hoechst 33342 generates CPD and induces accumulation of NER factor XPA. Cells irradiated with 405-nm laser with Hochst 33342 were co-immunostained with anti-CPD and anti-XPC antibodies. Bars, 20 μm (upper). GFP-XPA was accumulated at irradiated sites by the same treatment. Bars, 5 μm (bottom). (d) A rectangular region of interest (ROI) of a nucleus in shCtl, shDDB2 and shHBO1 cells was irradiated with a 405-nm laser. Representative pictures at indicated time points after irradiation are shown. Bars, 5 μm. (e) Depletion using different target sequences of siDDB2 and shHBO1 showed defective XPC-EGFP recruitment by laser irradiation. Cells were silenced by indicated shRNA or siRNA for 48 h. n=30 cells from three independent experiments; error bars are the mean ± s.e.m. (f) Stably expressing shHBO1-resistant Myc-HBO1 WT, EQ and SA cells that were depleted for endogenous HBO1. ShHBO1-resistant Myc-HBO1 constructs with three silent mutations. Stably expressing Myc-HBO1 WT, EQ and SA were detected at levels similar to endogenous HBO1 levels in the shCtl cells. (g) Recruitment of XPC-EGFP in Myc-HBO1 WT, EQ or SA expressing shHBO1 cells irradiated with a 405-nm laser. Representative pictures at indicated time points after irradiation are shown. Bars, 5 μm.

Supplementary Figure 4

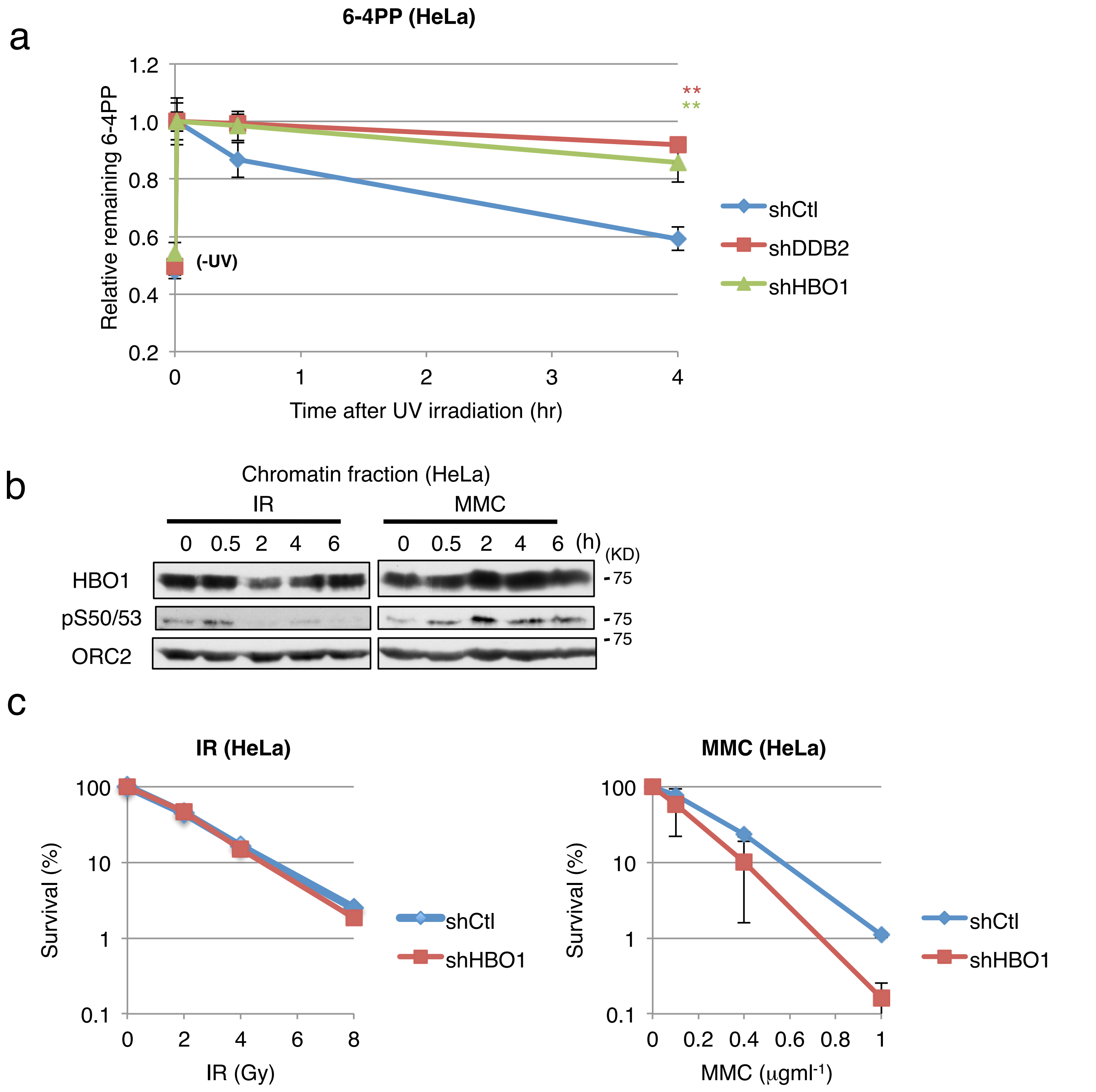

Supplementary Fig. 4 Defective capacity of 6-4PP repair in shHBO1 cells. Sensitivities to IR and MMC of shHBO1 cells.

(a) Depletion of HBO1 compromises repair of 6-4PP. ShCtl, shDDB2 and shHBO1 cells were not irradiated (-UV) or irradiated with 12 Jm^{-2} UV, cultured for 0, 0.5 and 4 h, and fixed and immunostained with anti-6-4PP antibody. DNA was counterstained with DAPI. Relative intensities of 6-4PP normalized against DAPI are shown as fold of UV 0 h. n=50 cells from three independent experiments; error bars indicate means ± S.D. ***P*<0.01 (Student's t test). (b) Phosphorylation of HBO1 pS50/53 in response to IR and MMC treatments. HeLa cells were irradiated with 10 Gy IR or pre-treated with 50 $ngml^{-1}$ MMC for 1 h and then washed with PBS. Cells were cultured for indicated times. Chromatin fractions of cell lysates were subjected to western blotting with indicated antibodies. Time at 0 h means no treatments. (c) Depletion of HBO1 sensitizes cells to MMC but not IR treatments. ShCtl and shHBO1 cells were irradiated with the indicated dose of IR or pre-treated with the indicated concentration of MMC for 1 h. Cells were cultured for two weeks. The numbers of colonies were counted and represented as % survival of mock treated cells. Five independent experiments were performed. Error bars indicate means ± S.D.

Supplementary Figure 5

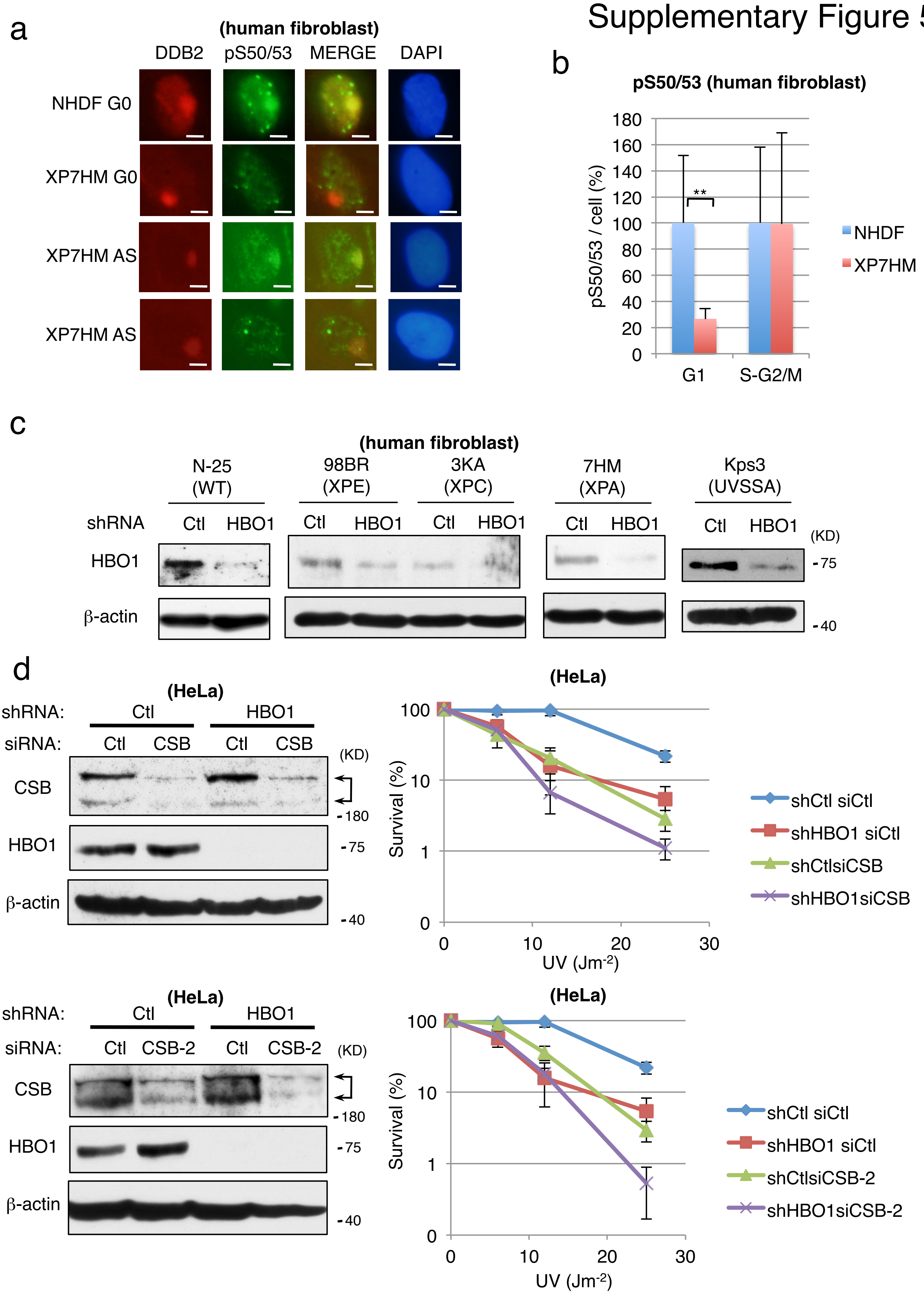

Supplementary Figure 5 continued

Supplementary Fig. 5 Establishment of HBO1-depleted primary fibroblasts from normal, various XP and UV^{S}S-A patients and double depletion of HBO1 and CSB in HeLa cells.
(a) Ser50 and Ser53 of HBO1were not phosphorylated in non-dividing XPA fibroblasts. NHDF (WT) or XP7HM (XPA) fibroblasts were cultured in serum starved (G0) or normal medium (asynchronized, AS) for 48 h. Cells were irradiated with 50 Jm^{-2} UV and cultured for 30 min. Cells were fixed and co-immunostained with anti-pS50/53 and anti-DDB2 antibodies. Bars, 5 μm. Phosphorylations of Ser50 and Ser53 of HBO1 were greatly diminished in G0 XP7HM cells, whereas AS XP7HM cells were either phosphorylated or greatly diminished phosphorylations of Ser 50 and Ser 53 of HBO1. Phosphorylated Ser50 and Ser53 of HBO1 were always detected in G0 NHDF cells. (b) Phosphorylation of Ser50 and Ser53 of HBO1 were diminished in XPA fibroblasts at G1 phase. AS NHDF or AS XP7HM fibroblasts were irradiated with 50 Jm^{-2} UV and cultured for 30 min. Cells were fixed and immunostained with anti-pS50/53 antibody. DNA contents were determined by DAPI staining to separate G1 and S-G2/M phases. Average of pS50/53 intensity in G1 and S-G2/M phase of each XP7HM cell was shown as % of NHDF cells. n=600, error bars indicate means ± S.D. ** $P<0.01$ (Student's t test). (c) To generate HBO1-depleted primary normal, XP and UV^{S}S-A fibroblasts, N25 (WT), 98BR (XPE), 3KA (XPC), 7HM (XPA) and Kps3 (UVSSA) cells were infected with shHBO1-B lentiviral particles following the manufacturer's instructions (Santa Cruz). Cells were selected with 10 μM puromycin for 3 days. Expression of HBO1 in each cell type was checked by western blotting. (d) Depletion of CSB and HBO1 additionally sensitized HeLa cells to UV irradiation. ShCtl or shHBO1 cells were transiently depleted for CSB by siCSB or siCSB-2 and clonogenic assays were performed as described in Methods. Six independent experiments were performed. Error bars indicate means ± S.D.

Supplementary Figure 6

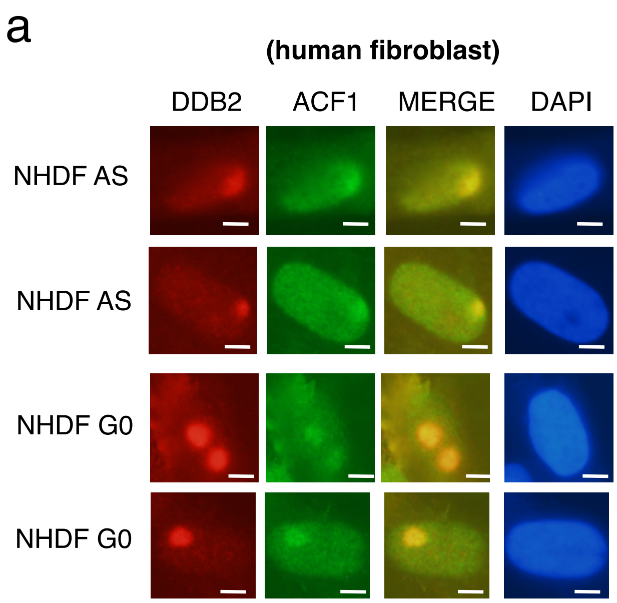

	AS	G0
G0/G1	52	70
S	24	18
G2/M	24	12

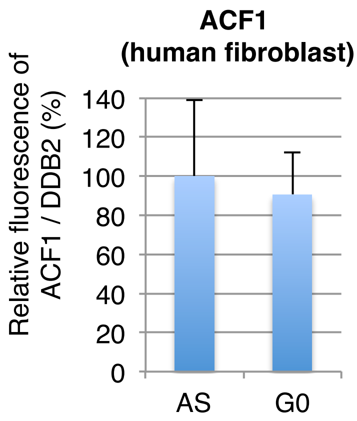

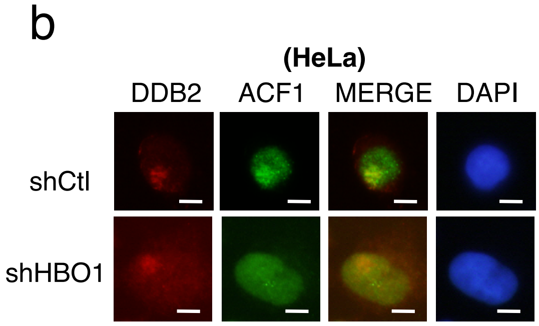

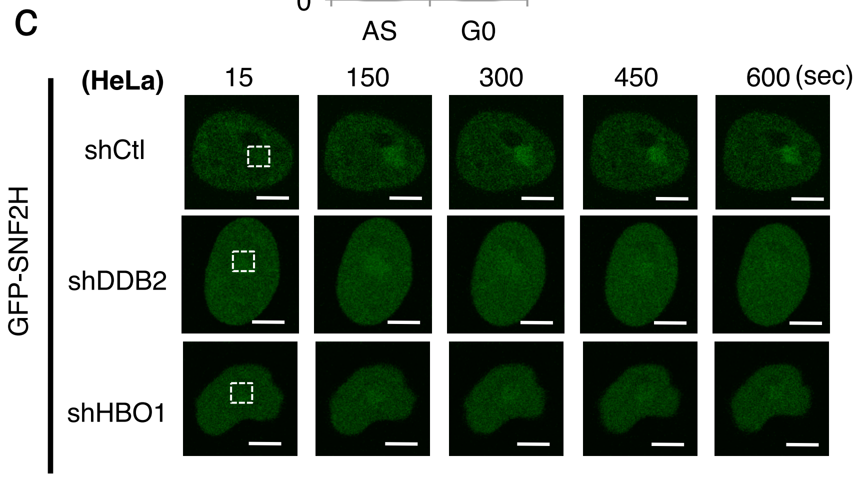

Supplementary Fig. 6 The ACF1-SNF2H chromatin remodeller accumulates at UV-induced DNA damage sites in an HBO1-dependent manner.

(a) ACF1 was accumulated at UV-induced damage sites in non-dividing cells. To explore whether accumulated ACF1 was not responsive to DSB after UV irradiation. NHDF cells were synchronized at G0 by serum starvation for 48 h, then irradiated with 50 Jm^{-2} UV and cultured for 30 min. Cell cycle of AS and G0 NHDF cells is shown in the table. Cells were co-immunostained with anti-DDB2 and anti-ACF1 antibodies. Definition of relative fluorescence was shown in Supplementary Fig. 1b. The relative ACF1/DDB2 intensity of G0 cell was shown as % of AS cells. Bars, 5 μm. (b) Accumulation of ACF1 was diminished in HeLa shHBO1 cells. Cells were irradiated with 50 Jm^{-2} and cultured for 30 min. Cells were fixed and co-immunostained with anti-DDB2 and anti-ACF1 antibodies. Relative intensity of ACF1 divided by DDB2 was shown in Fig. 7b. Bars, 5 μm. (c) Accumulation of GFP-SNF2H in 405-nm laser-irradiated areas. A ROI of a nucleus in shCtl, shDDB2 and shHBO1 cells was irradiated with a 405-nm laser with Hoechst33342. Representative pictures at indicated time points after irradiation are shown. Bars, 5 μm.

Supplementary Figure 7

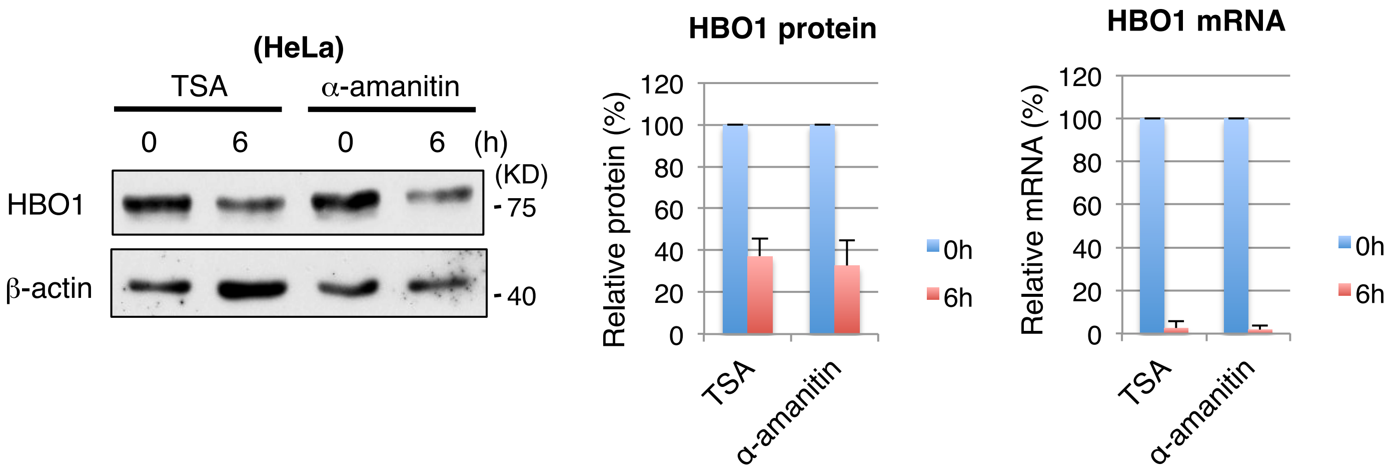

Supplementary Fig. 7 Inhibition of transcription and histone de-acetylation strongly inhibited expression of HBO1 mRNA and protein.
Cells were treated with 100 nM trichostatin A (TSA) or 25 μgml^{-1} a-amanitin for 6 h. Expressions of HBO1 mRNA and protein were measured by q-PCR and western blotting, respectively. Relative mRNA and protein expressions are represented as % of no treatments (0 h). Three independent experiments were performed. Error bars indicate means ± S.D.

Supplementary Figure 8

a

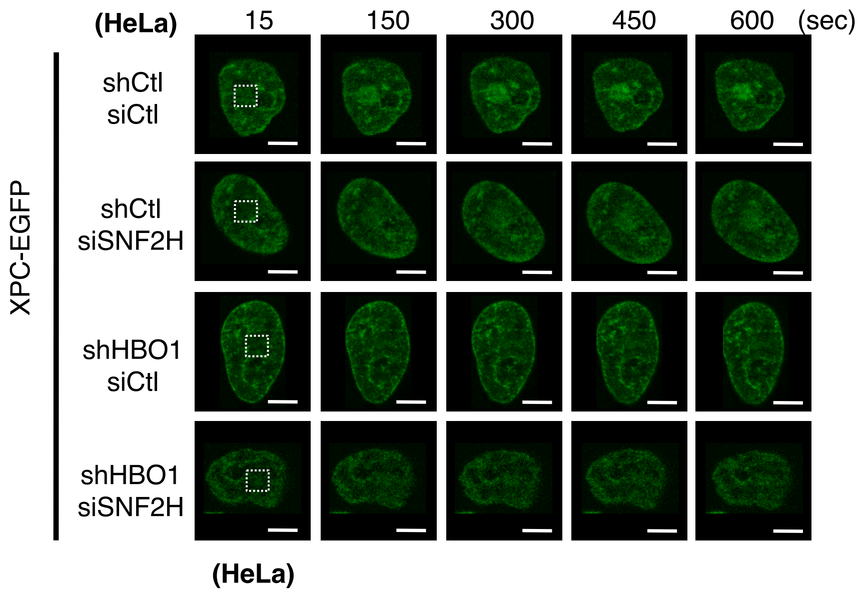

b

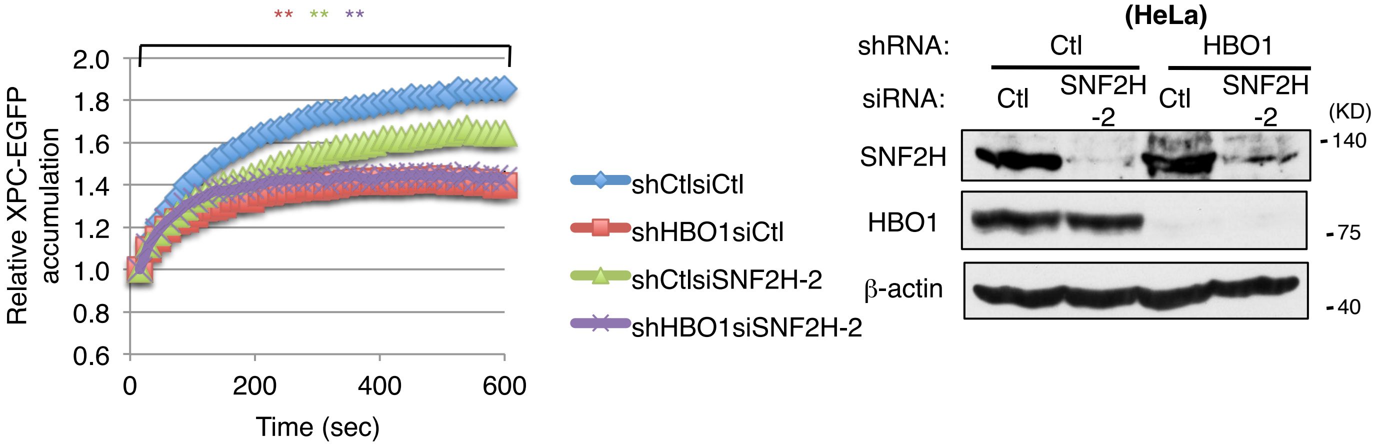

Supplementary Fig. 8 Involvement of SNF2H in accumulation of XPC at the damage sites.
(a) Accumulation of XPC-EGFP in 405-nm laser-irradiated areas. An ROI of a nucleus in shCtl siCtl, shCtl siSNF2H, shHBO1 siCtl and shHBO1 siSNF2H cells was irradiated with a 405-nm laser with Hoechst33342. Representative pictures at indicated time points after irradiation are shown. Bars, 5 μm. (b) Live cell imaging of XPC-EGFP accumulation at damage sites in cells depleted by siSNF2H (siSNF2H-2). XPC-EGFP and siCtl or siSNF2H-2 were transiently transfected in shCtl and shHBO1 cells. Laser irradiation and quantification of accumulated XPC-EGFP were performed as described in Methods. shCtlsiCtl n=75, shHBO1siCtl n=58, shCtlsiSNF2H-2 n=61, shHBO1siSNF2H-2 n=53 cells from at least six independent experiments; error bars are the mean ± s.e.m. ** P<0.01 (Student's t test).

Supplementary Figure 9

Uncropped immunoblots

Fig. 1b

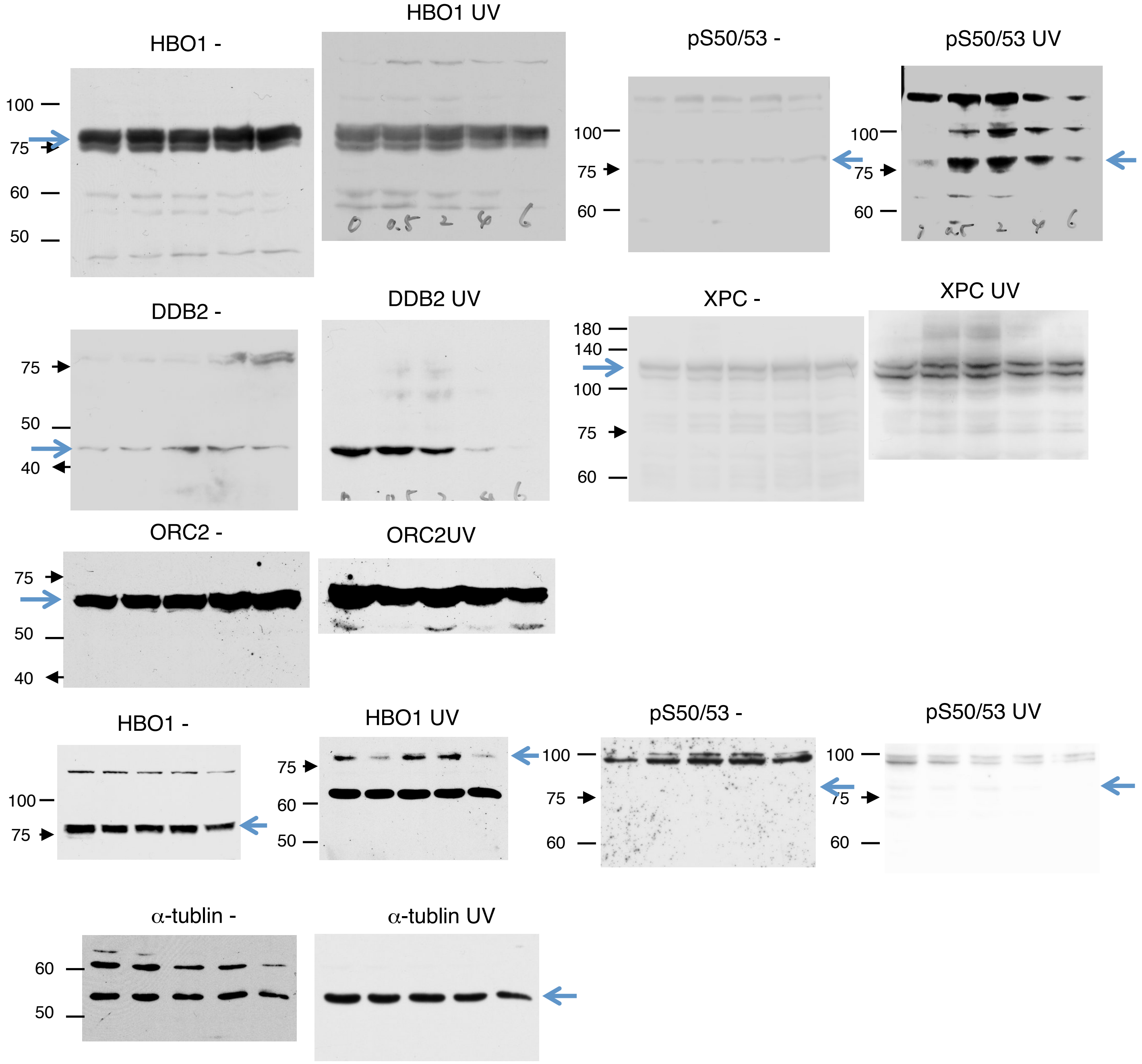

Supplementary Figure 9 continued

Fig. 2b

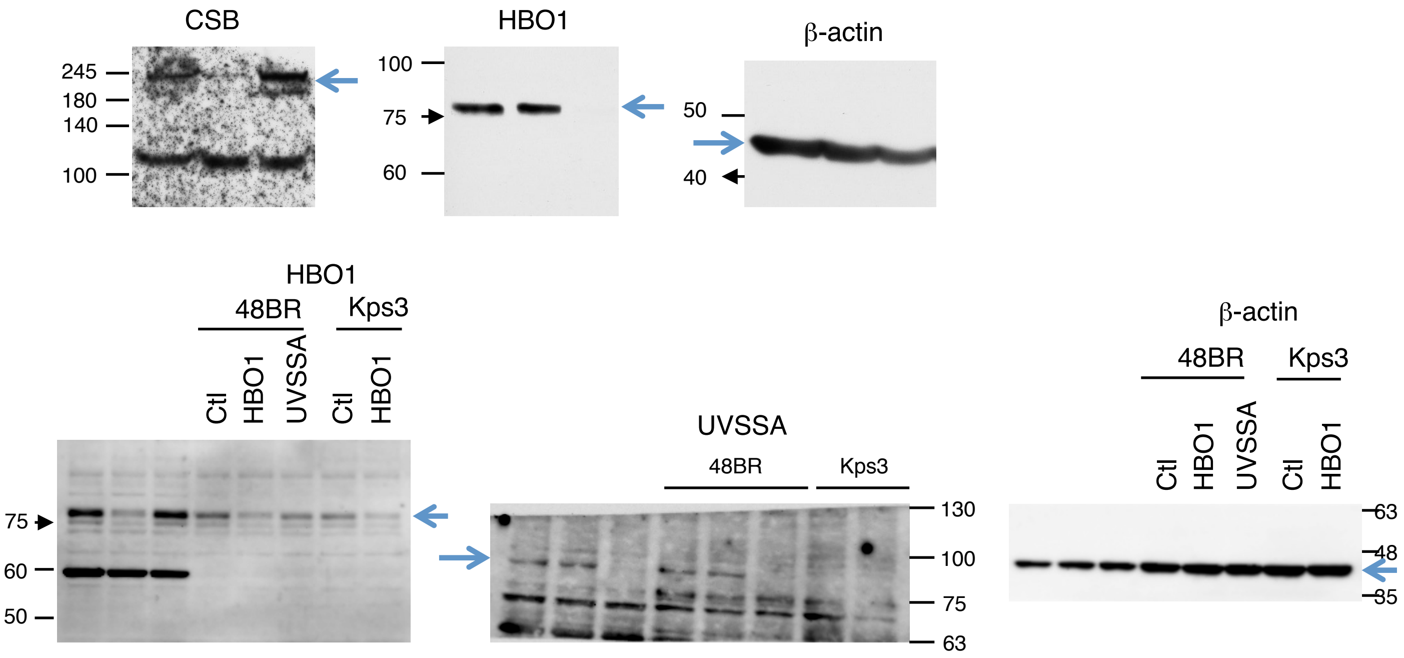

Fig. 4a

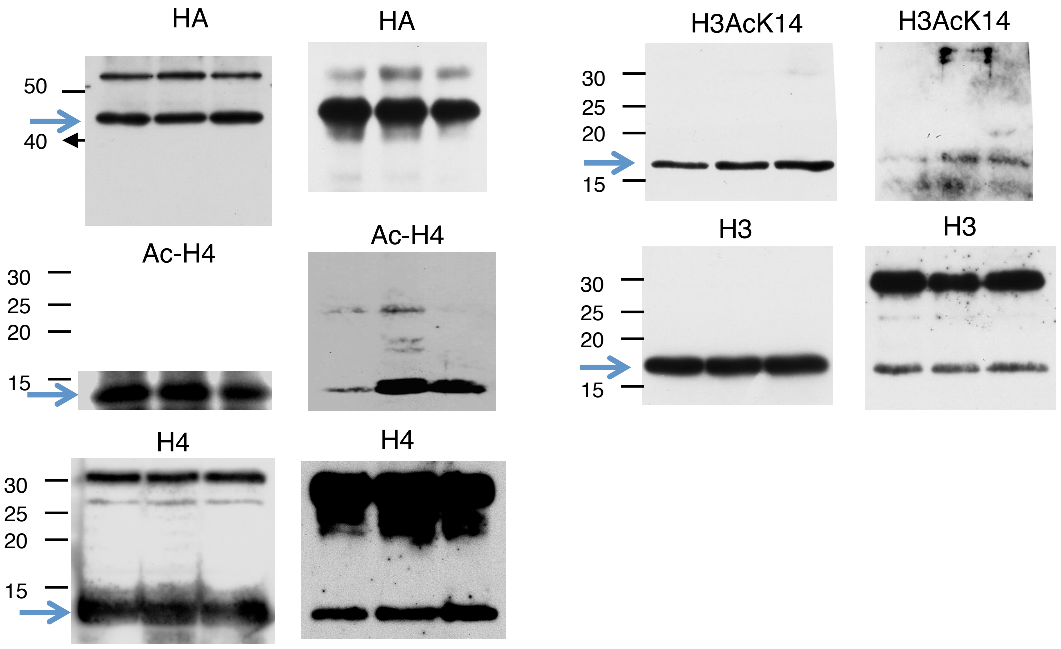

Supplementary Figure 9 continued

Fig. 4b

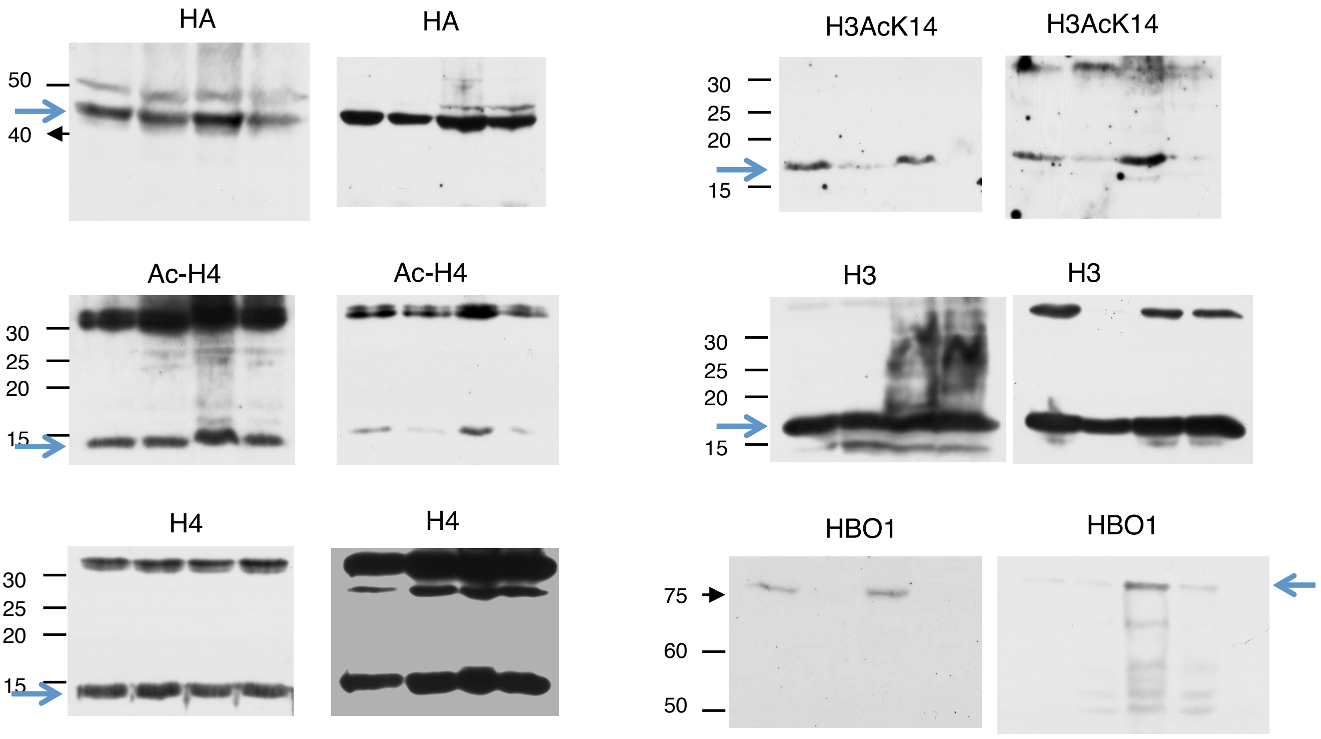

Fig. 7d

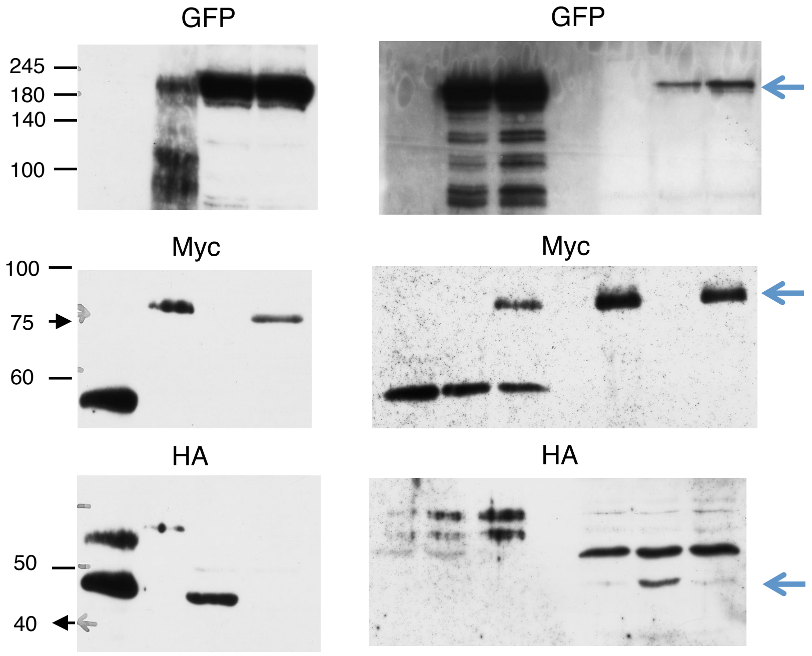

Supplementary Figure 9 continued

Fig. 7e

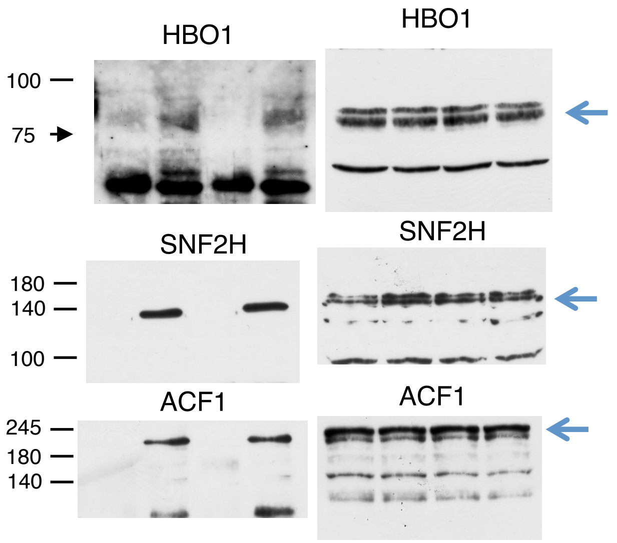

Fig. 7f

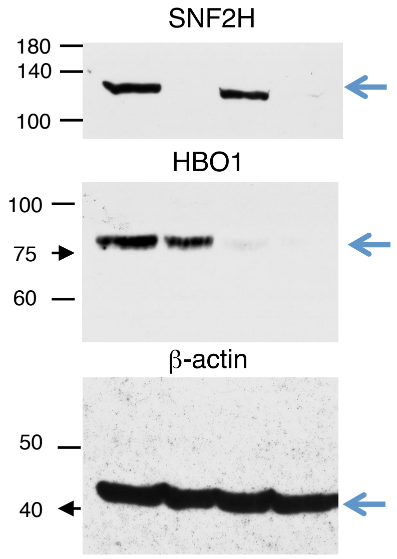

Fig. 7h

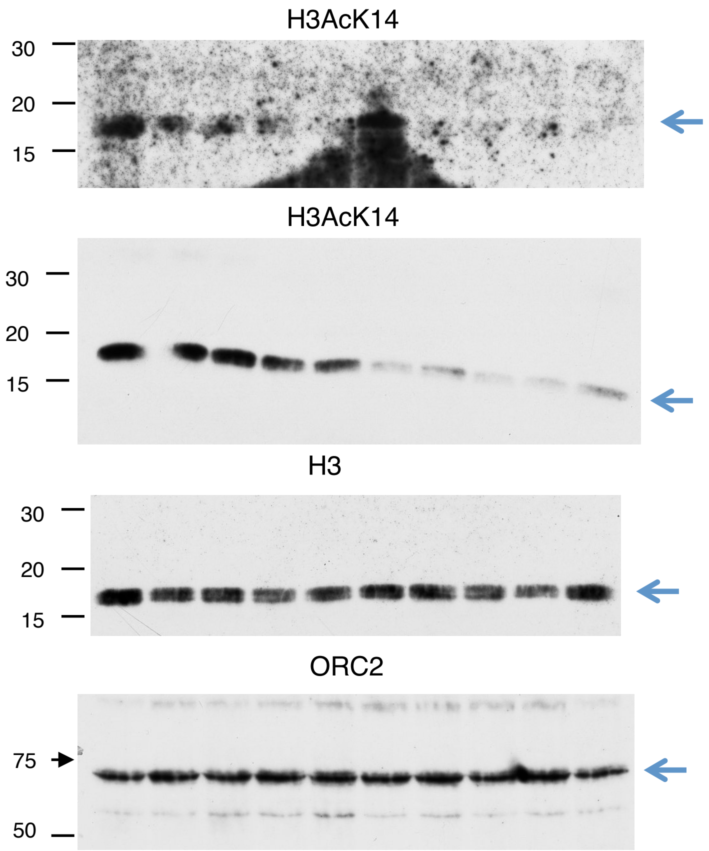

Supplementary Figure 9 continued

Supplementary Fig. 1a

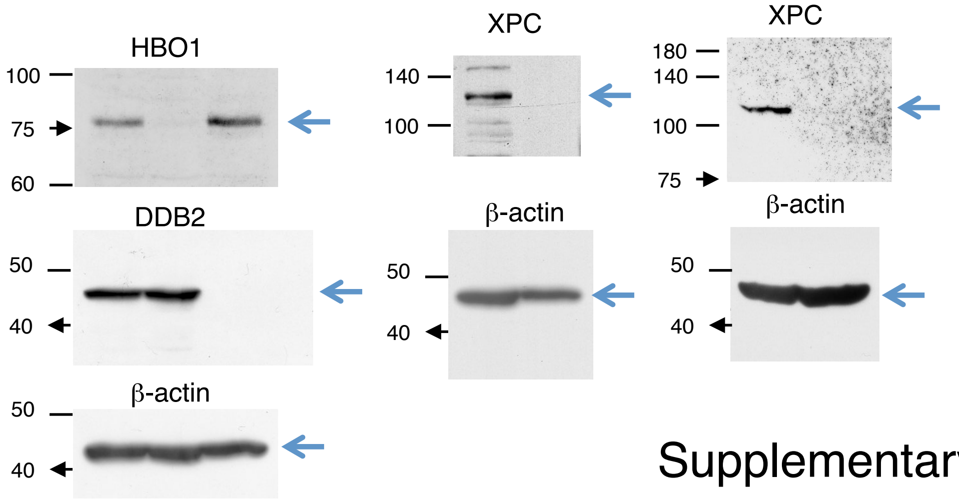

Supplementary Fig. 3f

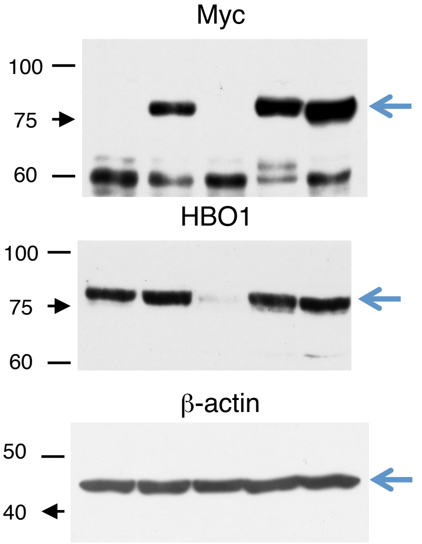

Supplementary Fig. 3e

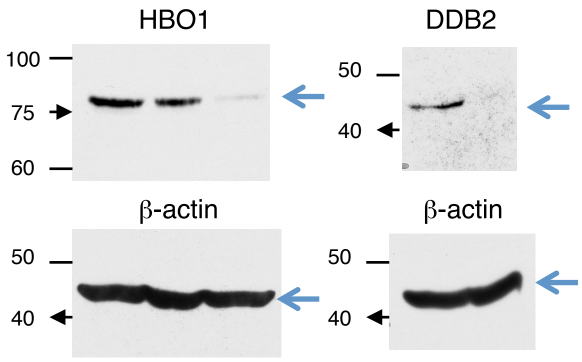

Supplementary Fig. 4b

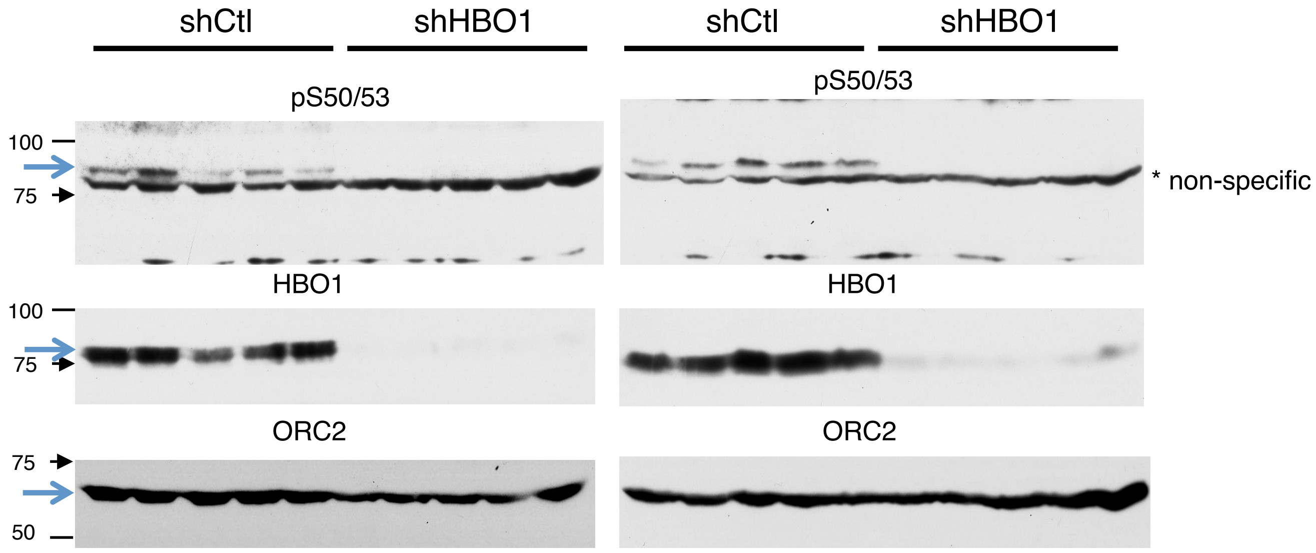

Supplementary Figure 9 continued

Supplementary Fig. 5c

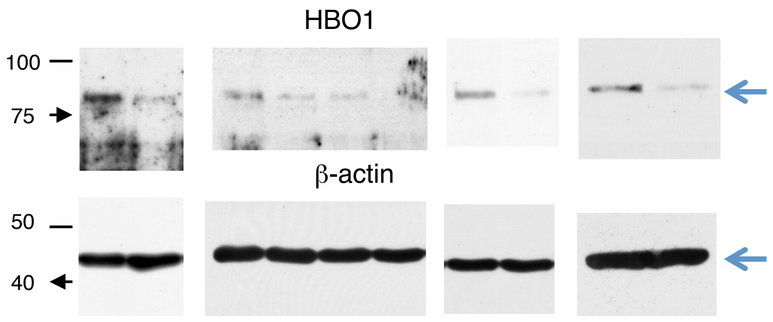

Supplementary Fig. 5d

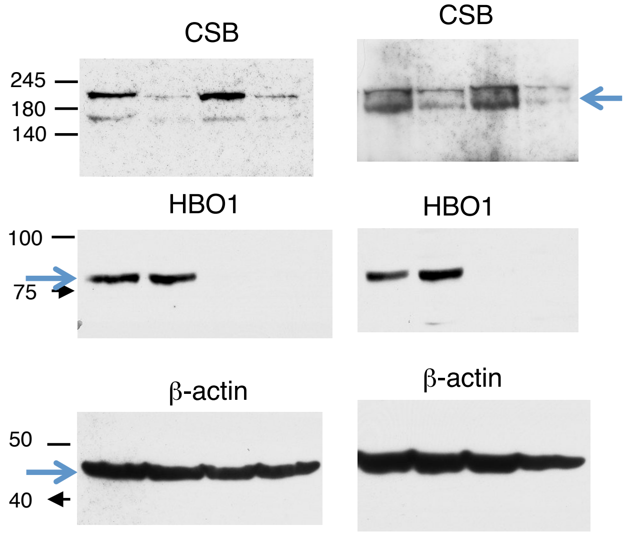

Supplementary Fig. 7

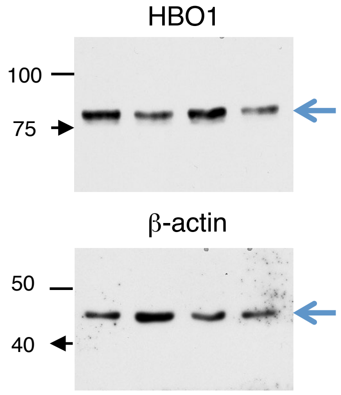

Supplementary Fig. 8b

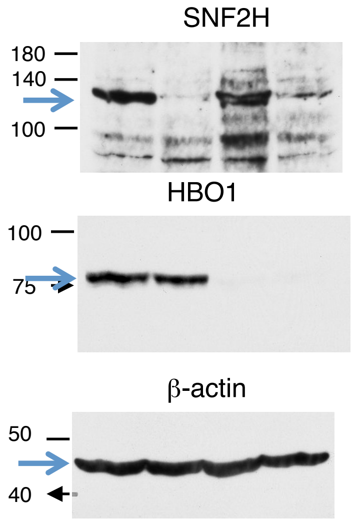

Supplementary Fig. 9 Uncropped immunoblots.
Uncropped immunoblots in figures and supplementary figures are shown.
